# Supplementary material for: The causal role of circulating inflammatory markers in osteoporosis: a bidirectional Mendelian randomized study
Source: Front Immunol. 2024 Jul 18;15:1412298. doi: 10.3389/fimmu.2024.1412298 (PMC11291241; doi:10.3389/fimmu.2024.1412298)
Supplement: Supplementary file 1 [file DataSheet_1.zip › supplementary material/OSTEOPOROSIS-CIM/GCST90274844.tsv.gz_leave_one_out.pdf]

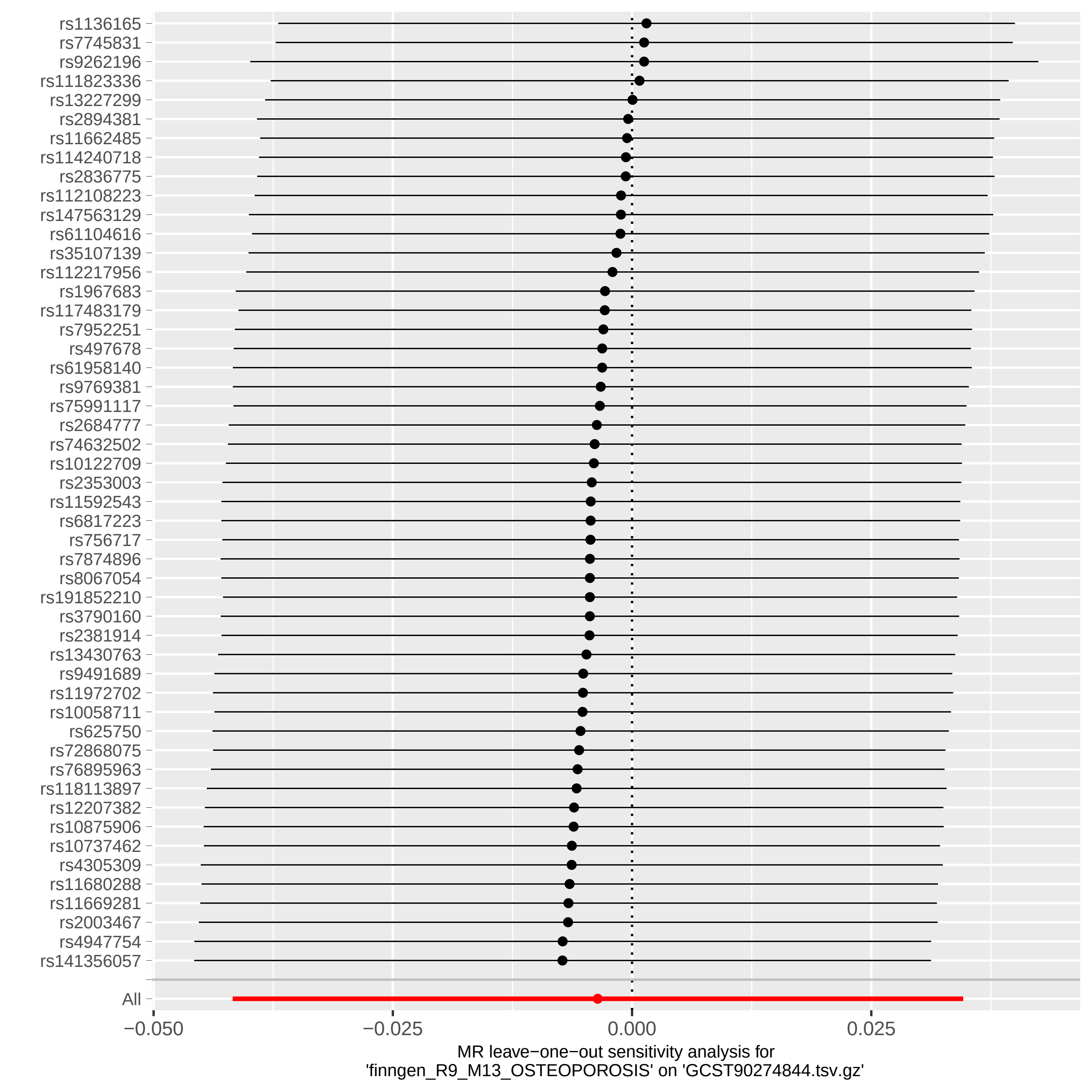
rs1136165
rs7745831
rs9262196
rs111823336
rs13227299
rs2894381
rs11662485
rs114240718
rs2836775
rs112108223
rs147563129
rs61104616
rs35107139
rs112217956
rs1967683
rs117483179
rs7952251
rs497678
rs61958140
rs9769381
rs75991117
rs2684777
rs74632502
rs10122709
rs2353003
rs11592543
rs6817223
rs756717
rs7874896
rs8067054
rs191852210
rs3790160
rs2381914
rs13430763
rs9491689
rs11972702
rs10058711
rs625750
rs72868075
rs76895963
rs118113897
rs12207382
rs10875906
rs10737462
rs4305309
rs11680288
rs11669281
rs2003467
rs4947754
rs141356057
All
−0.050
−0.025
0.000
0.025
MR leave−one−out sensitivity analysis for
'finngen_R9_M13_OSTEOPOROSIS' on 'GCST90274844.tsv.gz'
